# The Human Teeth*This short paper has been prepared in furtherance of the views expressed by the editor of the Register in a previous issue, and is expressly designed for publication in the newspaper press, and therefore, for non-professional readers only.

**Published:** 1860-01

**Authors:** 


### THE HUMAN TEETH.*

[* This short paper has been prepared in furtherance of the views expressed by the editor of the Register in a previous issue, and is expressly designed for publication in the newspaper press, and therefore, for non-professional readers only.]

"*Oh, that I had attended to my teeth before it was too late.*"

How many times has this, or similar expressions, been uttered, and with what earnestness? You, young farmer or farmer's daughter, how often when in young company, have you avoided a hearty laugh for fear of exposing your decayed teeth or toothless gums; how your pride has been touched, and your pleasures marred from this cause? Now you begin to appreciate the effects of the decay or loss of your teeth, but only in one particular—that of your personal appearance. But let us look farther, and see what evils follow, as decay progresses the utterance is changed; you lose the expression and emphasis, and the full tone of the natural voice; and still more, as you advance in life, your digestion becomes impaired, in consequence of your inability to properly prepare the food for the stomach, and then follows the train of evils attendant on "dyspepsia."

To epitomise,—the decay and subsequent loss of the teeth involves,

1st.—A foul mouth—disagreeable in appearance, and a constant source of annoyance to ourselves and those around us.

2d.—An impaired utterance—the loss of that peculiar quality, the full round ringing tone which is so attractive and the chief beauty and distinguishing trait of the human voice.

3d.—The labor and pain in attempts to masticate the food,—its imperfect preparation for the stomach—the digestive organs overtaxed, resulting in indigestion with a foul stomach and breath, and a host of evils, and lastly, the gradual change in the expression of the mouth terminating in the close proximity of nose and chin.

Now all these evils are the natural result of the neglect and loss of teeth. Would you avoid them? obtain at once professional advice, and you may be in time to have your teeth, though badly decayed, preserved to you for years, perhaps for life; or if your teeth be gone, have others substituted, which, though they can not be as useful as the natural organs, will relieve you to a very great extent from the troubles incident to the loss of your own teeth.

Now let me ask, in contemplating this picture, is there not sufficient inducement held out to avoid its evils? If so, the remedy is at hand, will you apply it? Yours,

*Philadelphia, December*, 1859. O. U. C.

---

PHILADELPHIA, Oct. 10, 1859.

MESSRS. EDITORS:—I inflict another desultory letter upon you. There is some instruction to be gained, I think, in watching the progress of the different new materials and methods brought to the notice of the profession.

*Gutta Percha* plates had quite a short life, and sadly disappointed the expectations of many honest and well meaning practitioners, the writer among the number.

*Cheoplasty* is still in use among a limited number, but I conclude is rather losing than gaining ground.

*Galvanism* in extractions seems to be dying out—to be falling into disuse.

All these had their earnest and honest supporters, and why? because of the hope that their was good in them, and from the earnest and sincere desire for improvement and progress.

*Vulcanite Rubber*, under its different titles and combinations, is rapidly coming into favor, and particularly in New York City, and throughout the State is being largely employed. Very intelligent dentists with whom I have conversed, many of whom have been using it for two years, (a pretty good test,) speak very flatteringly of it. The indications, therefore, are very favorable to its general adoption by the profession.

I have been led to say this much from conversations had with expert workmen in the mechanical department of dentistry, in this city and New York, who showed me some beautiful specimens of this work—very light, but remarkably strong, and who assured me that their business was rapidly increasing in this line, and from all they could learn from others and from their own experience, was led to the conviction that it afforded good satisfaction to the wearer, and answered to a great degree, all the purposes of an efficient artificial denture. Speaking of New York, I must tell you that dentistry there is in advance of "all the world and the rest of mankind," as I was assured by a member of the profession whom I met on a recent visit there. But joking aside, (which is not allowable in a high toned dignified Journal) they are an energetic people and all they lack is a spirit of harmony, by which, in an associated capacity, they may the better develop their strength.

What think you of a street dentistry? We have been favored for a brief period with a display of this sort in our city, in imitation of what may be seen in the French metropolis, viz: a person in the public street—on the wide pavement opposite our State House—with a chair and a few forceps and almost a peck of defunct molars and incisors, offering his services to a crowd of curiosity hunters, assuring them that "it don't hurt much, just set down here and I'll show you." Our people were not prepared for such a public exhibition, and want of encouragement, or the police, (I am not sure which,) drove the mountebank to a more suitable employment or another locality. Yours,

